# Emerging organoid models: leaping forward in cancer research

**DOI:** 10.1186/s13045-019-0832-4

**Published:** 2019-12-29

**Authors:** Han Fan, Utkan Demirci, Pu Chen

**Affiliations:** 1 Department of Biomedical Engineering, Wuhan University School of Basic Medical Sciences, 115 Donghu Road, Wuhan, 430071 Hubei China; 2 Hubei Province Key Laboratory of Allergy and Immunology, Wuhan, 430071 Hubei China; 3 Department of Radiology, Canary Center at Stanford for Cancer Early Detection, Stanford University School of Medicine, 3155 Porter Drive, Palo Alto, CA 94304 USA

**Keywords:** Cancer organoids, Patient-derived tumor organoids, In vitro model system, Cancer heterogeneity, Personalized anti-cancer therapy, Organ-on-a-chip, 3D Bioprinting

## Abstract

Cancer heterogeneity is regarded as the main reason for the failure of conventional cancer therapy. The ability to reconstruct intra- and interpatient heterogeneity in cancer models is crucial for understanding cancer biology as well as for developing personalized anti-cancer therapy. Cancer organoids represent an emerging approach for creating patient-derived in vitro cancer models that closely recapitulate the pathophysiological features of natural tumorigenesis and metastasis. Meanwhile, cancer organoids have recently been utilized in the discovery of personalized anti-cancer therapy and prognostic biomarkers. Further, the synergistic combination of cancer organoids with organ-on-a-chip and 3D bioprinting presents a new avenue in the development of more sophisticated and optimized model systems to recapitulate complex cancer-stroma or multiorgan metastasis. Here, we summarize the recent advances in cancer organoids from a perspective of the in vitro emulation of natural cancer evolution and the applications in personalized cancer theranostics. We also discuss the challenges and trends in reconstructing more comprehensive cancer models for basic and clinical cancer research.

## Introduction

Cancer leads to one in seven deaths worldwide. With the increase in the aging population, the global cancer burden is expected to rise to 21.7 million new cases and 13 million deaths by 2030, according to a recent WHO report [1]. While substantial progress has been made in standard anti-cancer treatment strategies, the effective treatments are still severely lacking primarily due to the tumor heterogeneity between and within individual patients. The tumor heterogeneity results in significant differences in the tumor growth rate, invasion ability, drug sensitivity, and prognosis among individual patients [2]. Therefore, the establishment of a high-fidelity preclinical cancer model is urgently needed to provide precise insights into cancer-related molecular evolution patterns in basic research and to allow personalized anti-cancer therapy in clinical.

Currently, immortalized cancer cell lines and patient-derived tumor xenografts (PDTXs) are commonly used in human cancer research. Cancer cell lines, which are characterized by low cost and ease of use, have been broadly employed in the high-throughput screening of drug candidates and cancer biomarkers. However, cancer cell lines can be only constructed from a limited number of cancer subtypes [3]. Moreover, the tumor-specific heterogeneity of cancer cell lines is gradually lost through epigenetic and genetic drift in the long-term culture [4]. In contrast, PDTXs retain tumor heterogeneity and genomic stability during the passage [5]. Besides, PDTXs can reproduce complex cancer-stroma and cancer-matrix interactions in vivo [6]. Nevertheless, the process of generating PDTX models usually takes more than 4 months, which may not be amenable for aiding terminal cancer patients. Additionally, PDTX models are expensive, labor-intensive, and incompatible with standard procedures in the high-throughput drug screening in the pharmaceutical industry (Table 1) [17–19].

**Table 1:** Advantages and disadvantages of using PTDX models and cancer organoids for cancer research

Feature	PDTX models	Cancer organoids
Generation efficiency	10%–70% [7, 8]	70%–100%
Tumor tissue source	Surgically resected specimens	Surgically resected or biopsy needle specimens
Retention of heterogeneity	Retention	Retention
Generation time	4–8 months	4–12 weeks [9–12]
Passage efficiency	Low	High
Genetic manipulation	Not amenable	Amenable
High-throughput screening for drug discovery	No	Yes
Immune components	Without	Retention [13–16]
Cost	High	Low

**Fig. 1:**
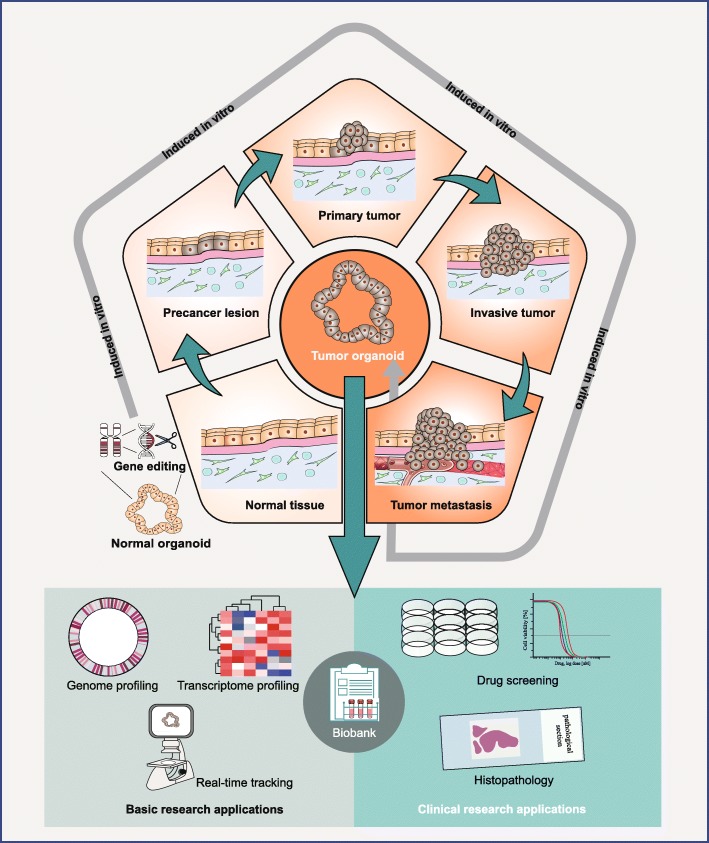
Cancer organoids can be derived from patients with diverse cancer grades and subtypes. Patient-derived organoids can possess patient-specific genetic and epigenetic contexts for preclinical cancer research and theranostics. Meanwhile, normal organoids can be used to model cancer evolution after the introduction of oncogenic mutations. By using the time-lapse microscopic imaging, tumor cell behaviors can be monitored in real-time. Similar to cell lines, cancer organoid lines can be expanded and cryopreserved to establish a living organoid biobank

Recently, the emergence of cancer organoid technology with the intrinsic advantage of retaining the heterogeneity of original tumors has provided a unique opportunity to improve basic and clinical cancer research [20]. The generation of cancer organoids is low cost, ease of use, and can be accomplished in around 4 weeks [21, 22]. Additionally, tumor organoid culture can be performed in the microplates which are compatible with standard high-throughput assays. Using the gene-editing technique, normal organoids can be mutated into tumor organoids, which may emulate genetic alterations during cancer initiation and progression. Currently, various patient-derived tumor organoids (PDTOs) have been generated, including liver, colorectal, pancreatic, and prostate cancer organoids (Table 2) [28, 29, 34, 35]. In this review, we provide an in-depth discussion of cancer organoids for basic cancer research, including carcinogenesis and cancer metastasis. Following this, we describe that the patient-derived cancer organoids offer a revolutionary approach for drug screening, immunotherapy, prognosis-related hallmark discovery. Finally, we conclude the pros and cons of cancer organoid and propose strategies for enhancing the fidelity of organoid in cancer research (Fig. 1).

**Table 2:** Cancer organoid models: published reports

Tumor organoid model	Cell derived	Research means	Achievement	Refs
Breast cancer organoids	Patient	Quantitative optical imaging	Predict the therapeutic response of anti-tumor drug in individual patients	[23]
	Mice	Organoid culture and xenotransplantation	Identify an early dissemination and metastasis mechanism for Her2+ breast cancer	[24]
Liver cancer organoids	Patient	Organoid culture and xenotransplantation	Establishment of hepatocellular carcinoma organoids from needle biopsies, and cancer organoids maintain the genomic features of the original tumors for up to 32 weeks	[11]
Gastric cancer organoids	Patient	Whole-genome sequencing	Identify mutated driver genes of promoting escape from anoikis in organoid culture	[25]
	Murine	Gene editing	First reveal the potential metastatic role of TGFBR2 loss-of-function in diffuse gastric cancer	[26]
Colorectal cancer organoids	Human stem cell	CRISPR-Cas9	Verify the deficient of key DNA repair gene MLH1 role in drives tumorigenesis	[27]
	Human stem cell	CRISPR-Cas9 and orthotopic transplantation	Visualize the different steps of the in vivo CRC metastatic cascade	[28]
Prostate cancer organoids	Patient, Mouse	Organoid culture and xenotransplantation	Show the role of nucleoporins in the progression of pancreatic cancer	[29]
	Patient	Organoid culture and xenotransplantation	Maintain prostate cancer-specific mutations and are suitable for in vitro and in vivo drug testing	[30]
Pancreatic cancer organoids	Patient	Organoid culture	The treatment profiles are parallel to the patient’s outcomes and the chemo-sensitivity of patient can be assessed	[31]
	Patient	Tumor organoids co-culture with stromal cells	Evaluate cancer-stroma cell interactions	[32]
Glioblastoma organoids	Patient	Organoid culture and xenotransplantation	Patient-derived organoids display histological features and recapitulate the hypoxic gradients in vivo	[33]

### Organoids for studying carcinogenesis

Carcinogenesis occurs through a temporal accumulation of cancer-specific genetic alterations in normal cells [36, 37]. However, the detailed process of genetic mutation in carcinogenesis is elusive. The in-depth investigation of these details is critical to understand nature carcinogenesis. Recently, researchers used a combination of organoid culture and CRISPR-Cas9 gene-editing technologies to add to this understanding. Matano, M. et al. demonstrated that targeting induction of driver pathway mutations in APC, SMAD4, TP53, KRAS, and/or PIK3CA in healthy human intestinal organoids could model the genesis of adenoma. However, these driver pathway mutations alone were not sufficient to induce colonic tumorigenesis [38]. Likewise, using lentiviral and retroviral infections, another group constructed oncogene-transformed organoids derived from healthy colon, stomach, and pancreas organoids. Consistent with previous clinical studies [39, 40], combinatorial genetic mutations of Kras^G12D^, p53, Apc, and Smad4 in healthy colonic organoids gave rise to adenocarcinoma organoids, while normal gastric and pancreatic organoids can be transformed into the adenocarcinoma organoids after p53 loss, Kras^G12D^ expression or both [41]. All these results demonstrated the utility of gene-edited organoid systems for the validation of the driver pathway mutations in tumorigenesis, thus providing a flexible in vitro cancer model for the study of tumorigenesis.

**Fig. 2:**
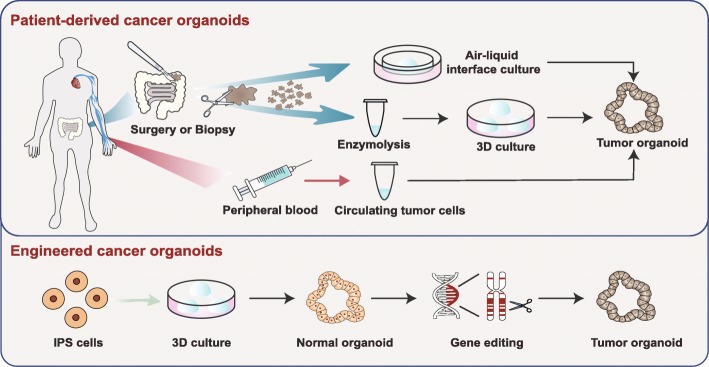
Patient-derived cancer organoids can be derived from surgically resected/biopsied tissues and circulating tumor cells. Additionally, using the gene-editing technique, normal organoids can be mutated into tumor organoids

Cancer organoid technology has also been used to investigate the complex interactions between genetic alterations and niche factors during carcinogenesis. For instance, Fujii, M. and his colleagues established colorectal cancer (CRC) organoids from endoscopic biopsies or surgically resected neoplasms of colorectal patients (Fig. 2). By screening the different combinations of niche factors in culture media, the researchers identified the niches that supported or inhibited the growth of CRC organoids. For example, CRC organoids that carried mutations in APC, CTNNB1, and TCF7L2 could grow without Wnt activators (Wnt3A/R-spondin1). The synergistic mutation of the KRAS gene and the PI3K pathway led to EGF independence in the growth of CRC organoids [42]. In general, cancer organoids with different carcinogenic mutations show distinct dependence on niche factors, providing an effective tool to understand the interaction between the genetic alterations and tumor microenvironment during carcinogenesis.

### Organoids for studying cancer metastasis

Cancer metastasis is a process of cancer cells spreading from the primary site to other organs, which contributes to the major cause of death in cancer patients. However, the underlying mechanisms driving metastasis are even more complicated than those resulting in carcinogenesis [43]. The ability to simplify the complexity and simultaneously retain the major pathophysiological features in the process is required to identify the critical factors in the acquisition of cancer metastatic potential. Cancer organoid has been increasingly used as a simplified and faithful in vitro model system to study cancer metastasis. Below, we describe the recent advances in applying cancer organoids to study cancer metastasis, including tumor invasion, metastasis, anoikis, and metastatic dormancy.

#### Tumor invasion and metastasis models

Predominantly, tumor invasion is regarded as a single-cell process. However, recent discoveries have implied that tumor invasion behaves as a cohesive multicellular unit, which is referred to as collective invasion [44]. Cancer organoids have been used as an optimizing model system to reveal the underlying mechanisms of collective invasion. For example, breast cancer organoids were used to investigate the role of leader cells that guide tumor cell invasion and intravasation. By using a live-cell microscopy assay, researchers found that BC organoids with the invasive phenotype extended multicellular strands of cancer cells into the extracellular matrix when the collective invasion was initiated by the specialized cancer cells that expressed K14 and p63 [45]. Similarly, by using cancer organoids, the researchers revealed that the cathepsin B led to the collective invasion in salivary adenoid cystic carcinoma [46], the inhibition of rho-associated protein kinase 2 (ROCK2) associated with initiating collective invasion in colorectal adenocarcinomas [47], and the loss of heat-shock factor 2 (HSF2) correlated with collective invasion in prostate cancer [48]. Moreover, extracellular matrix (ECM) in the tumor microenvironment, such as collagen I, could also modulate collective invasion in colon cancer organoids [49]. These studies exemplify that cancer organoid provides a trackable and reliable means to investigate tumor invasion.

Cancer organoids have also been used to identify the critical mutations that contribute to metastasis formation. Researchers have developed gene-edited CRC organoids carrying only the tumorigenesis driver pathway mutations APC, SMAD4, TP53, KRAS, and/or PIK3CA. These CRC organoids merely formed micrometastases when implanted into the spleen of mice. In contrast, the organoids with both chromosomal instability (CIN) and the tumorigenesis driver pathway mutations were capable of forming large metastatic tumors when transplanted into the mice [38]. These results suggested that CIN played an important role in modulating tumor cells to acquire metastatic behaviors in the CRC. In addition, cancer organoids could also aid in the discovery of critical targets for inhibiting tumor metastasis. In one study, Chandhoke, A.S. et al. discovered that the sumoylation of the PIAS3-Smurf2 pathway could inhibit the invasiveness of mammary tumor organoids [50]. Thus, the organoids provide an effective cancer model to study the mechanisms in promotion and inhibition of tumor invasion.

#### Tumor anoikis models

Anoikis refers to apoptosis of cancer cells induced by insufficient cancer-matrix interactions [51]. Anoikis resistance may allow the survival and proliferation of cancer cells and may contribute to tumor invasion and metastasis. Recently, intestinal organoids were used to study the effect of the RHOA mutation on the dissociation-induced apoptosis. Wang K. et al. genetically edited intestinal organoids with the RHOA mutations, which existed in approximately 14.3% of diffuse-type gastric cancer patients. Then, these organoids were dissociated into single cells. As expected, the RHOAmutation could lead to a higher efficiency of organoids recovery. More importantly, organoids carrying the RHOA mutation showed a better survival time and proliferative capacity, while the wild-type organoids were dead completely when without addition of the inhibitor of anoikis [25]. This result implied that the RHOA mutation could help cancer organoids escape from anoikis.

#### Tumor metastatic dormancy models

Metastatic dormancy is a leading cause of cancer recurrence [52]. However, the mechanisms of tumor metastatic dormancy and reactivation are still poorly understood. Cancer organoids have been demonstrated as a useful tool for tumor dormancy studies. Hattar, R. et al. demonstrated that tamoxifen could modulate cancer dormancy in a BC organoid model by reducing the fibronectin level in the extracellular matrix (ECM). BC organoids cultured on the tamoxifen-treated ECM displayed a smaller and smoother morphology compared to the BC organoids cultured on the tamoxifen-untreated ECM. Furthermore, they also found that tumor cell motility and invasion were suppressed by the tamoxifen treatment. These results were consistent with the previous clinical finding that increasing fibronectin level was associated with the lower survival rate in BC patients [53, 54]. Similarly, the antibodies to human collagen I can modulate the tumor dormancy in the BC organoid model by reducing the activity of collagen I in the ECM [55]. These results indicated that the ECM components in the tumor microenvironment could regulate tumor dormancy. In brief, cancer organoids can be used as a tool enabling effective screening of drug candidates that potentially prevent tumor recurrence.

### Patient-derived cancer organoids for personalized anti-cancer therapy

The therapeutic responses of anticarcinogens, especially for targeted drugs, strongly depend on the genetic and epigenetic contexts of cancer patients [56]. Although anticarcinogen discovery accounts for the highest proportion in the drug development market, the approval success rate for anticarcinogens is the lowest across the varied therapeutic areas. Moreover, even FDA-approved anticarcinogens display heterogeneous therapeutic responses and prognosis across individual patients [57]. Thus, it is critical to developing personalized anti-cancer therapy in screening drugs, optimizing immunotherapy, and discovering prognosis-related hallmarks.

#### Cancer organoid models for drug screening

Recent studies have demonstrated that PDTOs can capture the cancer-specific genetic alterations, gene expression, and histopathology in individual patients, which makes them suitable for personalized drug screening [9, 10, 30]. Sachs N and his colleagues constructed BC organoids from surgically resected specimens from 155 cancer patients. By comparing the therapeutic responses of anticarcinogen in the BC organoids and the corresponding patients, they found that the sensitivity to tamoxifen in the BC organoids was closely correlated with that in the original patients with metastatic BC [10]. More recently, personalized hepatocellular carcinoma organoids derived from needle biopsies were used to optimize drug dose for eight patients. The PDTOs displayed a distinct dose-dependent response to the sorafenib treatment in the different patients, which implied the potential value of PDTO models to predict patient-specific drug sensitivities to the targeted drugs [11]. Additionally, cancer organoids also act as an effective tool for interrogating gene-drug association. For example, Saito Y and colleagues constructed cancer organoids from surgically resected specimens from the patients with biliary tract carcinoma. They found that the TP53 mutant organoids were not sensitive to nutlin-3a, while the wild-type organoids were highly sensitive to nutlin-3a [12]. Similarly, the CRC organoids with the TP53 mutation was found insensitive to nutlin-3a [9]. These results agreed well with the clinical outcome in cancer patients with TP53 mutation.

#### Cancer organoid models for immunotherapy

Though the adoptive cell transfer and immunomodulatory checkpoint blockade have shown clear clinical benefit in the long-lasting anti-tumor immune responses, a large proportion of patients is insensitive to immunotherapy due to the heterogeneity of T cell repertoire and human leukocyte antigen (HLA) resulted from patient-specific neo-antigens [58–60]. Recent advances in tumor organoids offer a promising approach to generate tumor-reactive T cells. For example, Dijkstra KK et al. performed a coculture of tumor organoids with the patient’s peripheral blood lymphocytes. Under the stimulation of tumor organoids, tumor-reactive T cells with patient-specific immunogenic mutations were enriched and expanded, and then they could recognize and kill the autologous tumor organoids [13]. In addition, Finnberg NK et al. demonstrated that cancer organoids culturing at the air-liquid interface (ALI) could directly maintain the native tumor microenvironment for up to 44 days [14]. Furthermore, Neal JT and his colleagues indicated that the established tumor organoids using the ALI method could recapitulate the intrinsic tumor T-cell receptor spectrum and anti-PD-1/PD-L1-dependent human tumor-infiltrating lymphocyte (TIL) activation [15]. Meanwhile, cancer organoids have been used to study the effectiveness of combination immune therapy. Della Corte CM et al. investigated the efficacy of combining the anti-PD-L1 antibody with MEK inhibitor (MEK-I) or the anti-PD-1/PD-L1 therapy alone in non-small cell lung cancer (NSCLC) organoids. The research suggested that the combination therapies had a significantly higher drug response rate than the monotherapy owing to the increase of cell toxicity and immunoreactivity by the induction effect of MEK-I [16]. Notably, there are two clinical trials registered on the website of ClinicalTrials.gov, involving cancer organoids for immunotherapy (ClinicalTrials.gov number NCT03778814, NCT02718235).

Overall, these results indicate that cancer organoid culture is a promising system to generate tumor-reactive T cells, to predict immunotherapy sensitivity, and to examine combination immunotherapy.

#### Cancer organoid models for discovering prognosis-related hallmarks

Cancer organoids have been utilized as a platform to discover cancer prognosis-related hallmarks. Broutier L et al. discovered 30 potential tumor biomarkers by systematically comparing transcriptional differences between healthy organoid lines and primary liver cancer (PLC) organoid lines. Among these 30 tumor biomarkers, 19 genes were associated with PLC in clinical, and within 13 genes were related to poor prognosis in clinical. The researchers further analyzed the remaining 11 genes using The Cancer Genome Atlas (TCGA) and identified three genes associated with poor prognosis in hepatocellular carcinoma and one gene associated with poor prognosis in cholangiocarcinoma. Interestingly, STMN1 overexpression, which was previously thought to be associated with poor prognosis in only hepatocellular carcinoma, was proven here to be associated with low survival in cholangiocarcinoma in clinical [28]. These studies exemplify the potential value of PDTOs for tumor prognostic biomarker discovery.

#### Cancer organoid in clinical trials

The PDTOs provide a promising approach for personalized anti-cancer therapy in clinical. According to the studies registered on the website of the ClinicalTrials.gov as of November 1, 2019, there were 30 projects (1 terminated and 29 ongoing projects) related to cancer organoids. Among these trials, 53% were the observational studies and 47% belonged to the interventional studies, including one trial in phase I and five trials in phase II. Meanwhile, we noted that 73% projects aimed at studying anti-cancer therapy, including tailoring treatments for patients, identifying effective drug combinations, examining T-cell immunotherapy, and evaluating radiotherapy sensitivity; 13% projects aimed to generate patient-derived cancer organoid models; and the remaining projects focused on the mechanistic investigation of cancer onset and progression. Notably, these clinical trials involved a wide range of cancer types, including lung, pancreatic, prostatic, breast, esophagogastric, hepatocellular, biliary tract, neuroendocrine, and colorectal cancers, astrocytoma, and sarcoma [61].

In one clinical trial in the UK, Vlachogiannis G et al. carried out a phase I/II clinical trials to evaluate the clinical value of PDTOs in personalized anti-cancer therapy. In this trial, 71 patients with CRC or gastroesophageal cancer were recruited. Cancer organoids derived from patients’ biopsies displayed the 100% sensitivity, 93% specificity, 88% positive predictive value, and 100% negative predictive value, compared to the drug responses in the corresponding patients [62]. This study provided an encouraging proof that PDTOs can be employed as a clinically relevant model for anti-cancer therapy. Overall, we expect that the PDTO will revolutionize the conventional paradigm of anti-cancer therapy from systemic to individual approaches.

### Cancer organoid biobanks

Cancer organoid biobanks are repositories of PDTOs derived from diverse cancer grades and subtypes. In the repository, cancer organoids can be passaged and cryopreserved, just like immortal cell lines (Table 3) [30]. The establishment of cancer organoid lines can serve as a bioresource for fundamental and clinical cancer research due to several advantages of PDTOs, including cost-effectiveness, immediate accessibility, and proliferative capacity in vitro. Importantly, PDTOs display a much higher clinical relevance to their original patients than the immortal cancer cell lines. In addition, cancer organoid biobanks are more prominent for rare tumor subtypes that are difficult to generate stable lines. For instance, Sachs N and his colleagues established a BC organoid biobank, which had more than 100 common or rare cancer organoid lines derived from primary and metastatic BC [10]. Nevertheless, cancer organoid may lose their originally genetic and cellular heterogeneity during the long-term culture. By evaluating the genetic stability of a CRC organoid biobank containing 52 tumor subtypes, the researchers found that some organoid lines acquired new genetic mutations during the passage, especially in the microsatellite instability CRC organoids [42]. This result implied that the genetic stability of PDTO should be examined after passage to ensure the reliability of the research.

**Table 3.** Cancer organoid biobanks from various patients

Cancer types	Cancer organoid types in biobank	Success rate of establishment	Source	Passages	Institution	Refs
Metastatic gastrointestinal cancers	~78 metastatic cancer organoids from 71 patients	71%	Biopsies	Support	The Institute of Cancer Research, UK	[62]
CRC	22 cancer organoids from 27 tumor samples	~90%	Surgically resected	Support	Royal Netherlands Academy of Arts and Sciences, Holland	[9]
CRC	55 cancer organoids from 43 patients	100%	Biopsies, surgically resected	Support	Keio University, Japan	[42]
Breast cancers	> 100 cancer organoids from 155 tumors	>80%	Surgically resected	> 20 passages	Royal Netherlands Academy of Arts and Sciences, Holland	[10]
Pancreatic ductal adenocarcinoma	114 cancer organoids from 101 patients	75%	Biopsies, surgically resected, rapid autopsies	≥ 5 passages	Cold Spring Harbor Laboratory, America	[31]

### Future directions and opportunities

Although cancer organoid models resemble some critical features of human cancer development and progression, there are still plenty of spaces to improve the pathophysiological and clinical relevance of cancer organoids to tumors in situ further. Firstly, tumor organoids usually comprise only epithelial cell types and progenitor cells, but they do not contain nonparenchymal cell types such as fibroblasts and endothelial cells. Secondly, tumor organoid culture usually reconstitutes tumors in a single organ, but they cannot recapitulate cancer metastasis in the multiorgan. Additionally, conventional cancer organoid culture does not allow precise spatiotemporal control over biophysical and biochemical factors in the tumor microenvironment. The recent tendency in the synergistic application of organoid with organ-on-a-chip and 3D bioprinting enables to develop more sophisticated cancer models to study underlying mechanisms of tumor-stroma interactions, tumor multiorgan metastasis as well as cancer-microenvironment interactions.

#### Organoid-on-a-chip

A notable strategy is to generate organoid-on-a-chip by combining organoid with organ-on-a-chip. Organ-on-a-chip is a microfabricated device with integrated living cells, ECM, and microstructures to emulate partial aspects of organ or tissue in their cytoarchitecture, cellular population, and functions [63]. Organ-on-a-chip is featured with the capacities for precise microenvironment control, continuous flow perfusion culture, and high-throughput format. Notably, organ-on-a-chip allows integration of multiple mini-organs in the different microchambers interconnected via microfluidic channels to form human microphysiological system, which provides a unique platform to study cancer multiorgan metastasis via the circulatory system. Nevertheless, at present, most of the organ-on-a-chip systems utilize primary cell lines or stem-cell-derived cells as the cell source to construct organ mimics, and they cannot emulate histological and cellular complexity of native organs and tumors [64]. By incorporating multiple organoids into organ-on-a-chip, organoid-on-a-chip can inherit the benefits from both organoid and organ-on-a-chip and provide an effective tool to study tumor multiorgan metastases and cancer-microenvironment interactions.

**Fig. 3:**
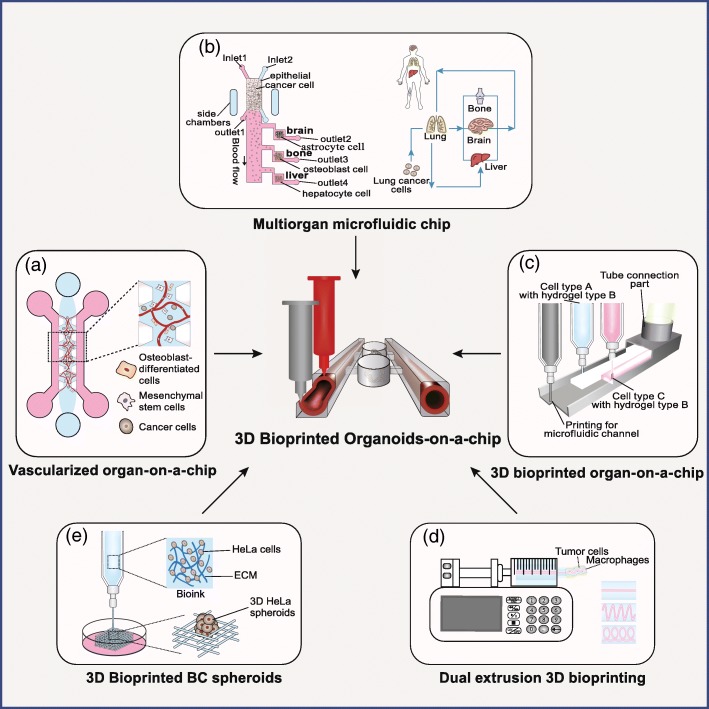
**a** A vascularized organ-on-a-chip model was utilized to analyze BC cell invasion and metastasis through a microvascular network. **b** A multi-organ-on-a-chip system was composed of a “primary site” and three “main sites of metastatic”. This microfluidic system was used to model lung cancer metastasis to distant organs, which provided an experimental platform to analyze cell-microenvironment interactions in organ-specific metastasis. **c** Schematic diagram of the 3D bioprinting technology for organ-on-a-chip models. **d** An extrusion-based bioprinting platform that interrogates the paracrine loop between BC cells and macrophages in different geometric arrangement. An extrusion-based 3D bioprinting technique for constructing breast cancer metastasis model. **e** Fabrication of the 3D HeLa/hydrogel spheroids by 3D printing

A 3D vascularized tumor model was constructed on a chip to study the mechanism of multiorgan metastasis from breast cancer (Fig. 3a). In this chip, endothelial cells (ECs), mesenchymal stem cells (MSCs), and osteoblast-differentiated cells (OBs) were cultured in 3D ECM to mimic bone marrow and muscle microenvironments with the microvascular networks. Extravasation rates of these metastatic BC cells were investigated on these microenvironments with or without adenosine treatment. The result showed that metastatic BC cells displayed distinct extravasation rates in different microenvironments, and blockage of A_3_ adenosine receptor in BC cells resulted in increased extravasation rate in the muscle microenvironment [65]. In another study, a four-organ-on-a-chip system was developed to model metastasis of primary lung cancer to the downstream organs, including the brain, liver, and bone (Fig. 3b) [66]. The results implied the metastasis displayed spatiotemporal heterogeneity over the different organs and ultimately led to the damages on all these four organs. However, these tumor-on-chip models were built with cancer cell lines and could not represent the critical features of the native tumor. In turn, incorporation of metastatic tumor organoids with other normal organoids on a chip presents a better way for studying cancer multiorgan metastasis.

#### 3D Bioprinting of organoid culture system

Another strategy is to develop sophisticated organoid culture systems for multiple tumor types by using 3D bioprinting. 3D bioprinting allows precise control over spatial heterogeneity in the tumor microenvironment by spatially deterministic deposition of predefined biobanks that may contain multiple cell types, biochemical factors, and ECM (Fig. 3c) [67–70]. For example, Grolman, J.M., et al. constructed a BC microenvironment to study the role of paracrine signaling network in the regulation of breast cancer metastasis. Breast adenocarcinoma (MDA-MB-231) and macrophages (RAW 264.7) were printed in the hydrogels with distinct spatial distributions and variable geometrical shapes by extrusion-based 3D bioprinting technique (Fig. 3d) [71]. The results indicated that geometric cues regulated the paracrine loop between BC cells and macrophages, which further initiated BC tumor intravasation into the bloodstream. Another example of an in vitro cervical tumor model was established to demonstrate the epithelial-to-mesenchymal transition (EMT). The HeLa cells were mixed with hydrogel and further be fabricated into cell-biomaterial constructs with grid shape by employing an extrusion-based 3D bioprinter (Fig. 3e) [72]. The results implied the supplement of TGF-β-induced EMT and this promoting effect was inhibited by the treatment of disulfiram and EMT pathway inhibitor C19 in a dose-dependent manner, which suggested that the tumor metastasis in 3D culture was a comprehensive result involving the complex interacions between tumor cells, ECM, and 3D microenvironment.

## Conclusion

Cancer organoids exhibit higher physiological and clinical relevance than cancer cell lines and animal cancer models. Meanwhile, the PDTOs can effectively retain the molecular, cellular, and histological phenotypes of original cancer patients and maximally maintain patient-specific tumor heterogeneity compared to the common cancer cell lines and PDTX models. Therefore, cancer organoid models provide a powerful tool for advancing our understanding of tumor evolution and have great clinical significance in personalized anti-cancer therapy. Furthermore, synergistic applications of organ-on-a-chip and 3D bioprinting to organoids present a new trend to achieve more comprehensive cancer model systems, enabling precise regulation of tumor microenvironment, incorporation of microvascular network, and integration with multiple organs. Overall, we expect that these emerging in vitro cancer model systems will ultimately revolutionize the conventional paradigm of cancer research and produce true benefits in clinical.

## Data Availability

Not applicable.

## Abbreviations

3D: Three-dimensional
ALI: Air-liquid interface
BC: Breast cancer
CIN: Chromosomal instability
CRC: Colorectal cancer
ECM: Extracellular matrix
ECs: Endothelial cells
EMT: Epithelial-to-mesenchymal transition
FDA: U.S. Food and Drug Administration
HLA: Human leukocyte antigen
HSF2: Heat-shock factor 2
MEK-I: MEK inhibitor
MSCs: Mesenchymal stem cells
NSCLC: Non-small cell lung cancer
OBs: Osteoblast-differentiated cells
PDTOs: Patient-derived tumor organoids
PDTXs: Patient-derived tumor xenografts
PLC: Primary liver cancer
ROCK2: Rho-associated protein kinase 2
TCGA: The Cancer Genome Atlas
TIL: Tumor-infiltrating lymphocyte
WHO: World Health Organization
